# Effect of monovalent electrolyte solutions on the human tear ferning pattern

**DOI:** 10.1371/journal.pone.0280853

**Published:** 2023-02-03

**Authors:** Essam S. Almutleb, Gamal A. El-Hiti, Hesham A. Al-Dawas, Mohammed K. Alanzi, Mohammed Alquwayi, Abdullah G. Alotaibi, Mashaaer A. Baashen, Basal H. Altoaimi, Saud A. Alanazi, Ali M. Masmali

**Affiliations:** Department of Optometry, College of Applied Medical Sciences, King Saud University, Riyadh, Saudi Arabia; Tribhuvan University, NEPAL

## Abstract

**Purpose:**

To investigate the effect of the addition of a low concentration of sodium chloride (NaCl) and potassium chloride (KCl) solutions on the tear ferning (TF) patterns of tears collected from humans.

**Methods:**

A tear sample (20 μL) was collected from the right eye of 23 males and 7 females (25.4 ± 6.6 years). The tears were collected in one sitting for healthy subjects (*N* = 13). For dry eye participants (*N* = 17), the tear samples were collected in two separate settings with five minutes gap in between. A sample (1 μL) from each tear was dried on a glass slide, and the obtained ferns were observed using a microscope and graded using the five-point TF grading scale. Mixtures of tear samples (0.5 μL) and different volumes (0.5–2.5 μL) of each electrolyte (10–30 mg in 100 mL of water) solution were prepared, and their TF patterns were recorded and compared with those of the corresponding pure tears.

**Results:**

Significant improvements (Wilcoxon test, *P* < 0.001) have been seen in the TF grades of the tear samples after the addition of NaCl and KCl solutions. A significant difference (Wilcoxon test, *P* = 0.016) was found between the TF grades when NaCl and KCl solutions were added to the tear samples. The TF grades of pure tears collected from dry-eye subjects ranged from 2.1 to 3.5, based on the five points grading scale, and decreased to be in the range of 0.4 to 1.6 after the addition of electrolyte solutions. While the TF grades of pure tears collected from normal-eye ranged from 1.2 to 1.9 and improved after the addition of electrolyte solution to be in the range of 0.4 to 1.5.

**Conclusions:**

The TF test was used *in vitro* to assess the impact of the addition of a low concentration of sodium and potassium chloride solutions on tears collected from humans. The TF grades of human tears significantly improved after the addition of either sodium or potassium chloride solution. The mechanism for the improvement in TF grades due to the addition of electrolyte solutions must be investigated.

## Introduction

The stability and quality of the tear film are vital for the health of the ocular system. The function of the tear film includes the lubrication of the ocular surface, protection against microorganisms, and the washing of debris and foreign bodies [1]. Dysfunction in tear film causes disturbance in vision and leads to several ocular diseases such as dry eye. Dry eye is a common ocular disorder characterized by the loss of homeostasis of the tear film [2]. Dry-eye symptoms vary across individuals, but the most common ones include discomfort, pains, redness, inflammation, and watery eye [3, 4]. The prevalence of dry eye increases, and around 33% of the world population suffer from such disorder [3, 4]. Many factors contribute to dry eye; however, meibomian gland dysfunction is one of the most common causes of eye dryness. It leads to an alteration in the tear film lipid layer and therefore an excessive evaporation of tears. Meanwhile, insufficient tear production leads to a hyposecretory dry eye [5]. Other common risk factors for dry eye include diabetes, thyroid gland disorder, high body mass index, refractive errors, smoking, harsh environments (e.g., high humidity and temperature), vitamin A and D deficiencies, contact lens wearing, and ocular surgeries. Dry-eye diagnosis is a challenge, and no single test can provide a definite result in which various parameters (e.g., volume, quality, osmolarity, and stability of tears) should be assessed. Therefore, it is recommended to use a combination of invasive and noninvasive tests for the proper diagnosis of dry eye. The common tests for dry-eye diagnosis include the assessment of osmolarity [6], noninvasive tear breakup time [7], symptoms [8], and stinging [9]. The volume of the tears can be assessed using tear meniscus height [7], Schirmer [10], and phenol red thread [10] tests. The rate of evaporation of tears can be measured using the tear evaporation rate test [11, 12]. The tear ferning (TF) test can be used to assess the quality of tears [13]. In addition, objective methods have been used for tear fern analysis [14].

The TF test can be used as a repeatable *in vitro* method to assess the quality of tears collected from humans and animals [15–18]. Tears, when dried, produce crystals in specific patterns known as ferns. High levels of humidity and temperature deteriorate the ferns and therefore should be avoided in the drying process [19]. The precipitation of large molecules such as proteins and salts including sodium chloride (NaCl) and potassium chloride (KCl) in tears is responsible for the production of ferns [20]. Therefore, the TF patterns changed upon the addition of electrolytes or large molecules to tears. These changes could lead to a disturbance in the stability and composition of the tear film. Tears can be collected from humans or animals in different ways, but the use of capillary tubes is the simplest and most common [21]. Two grading scales are commonly used to assess the ferns of dried tears. The first one involves grades I and II for normal eyes and III and IV for dry ones [22]. The second one involves the use of five grades (0–4) and is used in 0.1 increments. A grade below 2 is indicative of normal and healthy tear film [23].

In recent years, the effect of the addition of electrolyte solutions on the TF patterns of human, animal, and artificial tears was investigated [24–27]. However, various limitations have been associated with these reports, such as low sample size, lack of statistical analysis, and limited or no improvements in TF grades after the addition of NaCl and KCl solutions. Therefore, the current study was conducted to overcome some of the limitations associated with the previous related report [27]. We report an improvement in TF grades of tears collected from humans after the addition of a low concentration of NaCl and KCl solutions.

## Materials and methods

### Study design, subjects, and ethics

This observational nonrandomized comparative in-vitro study was performed at the clinics at the College of Applied Medical Sciences, King Saud University. Thirty subjects (23 males and 7 females) who ranged from 18 to 39 years old (25.4 ± 6.6 years) were recruited to collect tear samples in Riyadh City. The participants were classified as healthy (*N* = 13) and dry eyes (*N* = 17) based on the TF grades of their pure tears. The study was approved by the IRB at King Saud University (E-22-6562). Informed written consent was obtained from the subjects before collecting the tears.

### Electrolyte solutions

The slats (NaCl and KCl) were obtained from Avonchem Limited (Wellington House, Macclesfield, UK). Each slat (10–30 mg) was dissolved in double-distilled water (100 mL), and the solutions were stirred for 5 minutes to ensure complete solubility.

### TF test

Glass capillary tubes (50 μL) were purchased from Sigma-Aldrich Chemical Company (Gillingham, UK) to collect the tears. A tear sample (20 μL) was collected from the right eye of each subject. A sample from each tear (1 μL) was dropped on a microscopic slide and left to dry (10 minutes at 22°C and 15% humidity). The tears were collected in one sitting for healthy subjects (*N* = 13). For dry eye participants (*N* = 17), the tears were collected in two separate settings with five minutes gap in between. Dry eye was defined for a TF grade of TF ≥ 2 based on the five-point grading scale [23]. The TF grade of the tears collected from the same subjects in two different settings was similar. The ferns of the dried tears were viewed using an Olympus DP72 light microscope (Olympus Key Med Ltd., UK). The ferns were graded in 0.1 increments by two independent researchers using the five-point TF grading scale [23]. Mixtures containing human tears (0.5 μL) and NaCl or KCl solutions (10–30 mg, 0.5–2.5 μL) were obtained. The proportions of tears to electrolytes were 1:1, 1:2; 1:3, 1:4, and 1:5 for 10 mg in 100 mL of water. For the solutions containing 20 or 30 mg of NaCl or KCl in 100 mL of water, the proportions of tears to electrolyte were 1:3, 1:4, and 1:5. A sample of each mixture (1 μL) was dried on a glass slide at standard conditions, and the ferns produced were observed and graded. The TF grade of each mixture was compared with those for the corresponding pure human tears. The dilution effect on the TF patterns of tears was tested in which different proportions of water were added to the tear samples (1:1 to 1:5). No changes were noticed in the TF grades of tears after the addition of water, which is consistent with a previous report [27]. Two masked examiners graded the TF patterns.

### Statistical analysis

Excel (Microsoft Office 2016, Microsoft Corp., Redmond, WA, USA) was used to record the data. Statistical Package for the Social Sciences (SPSS) (IBM Software, version 25, Armonk, NY, USA) was used to analyze the data. The data were not normally distributed (Shapiro–Wilks test, *P* < 0.05). The significance (Wilcoxon test, *P* < 0.05) of the improvement in TF grades of human tears after the addition of NaCl and KCl solutions was tested using the Wilcoxon test.

## Results

**Fig 1:**
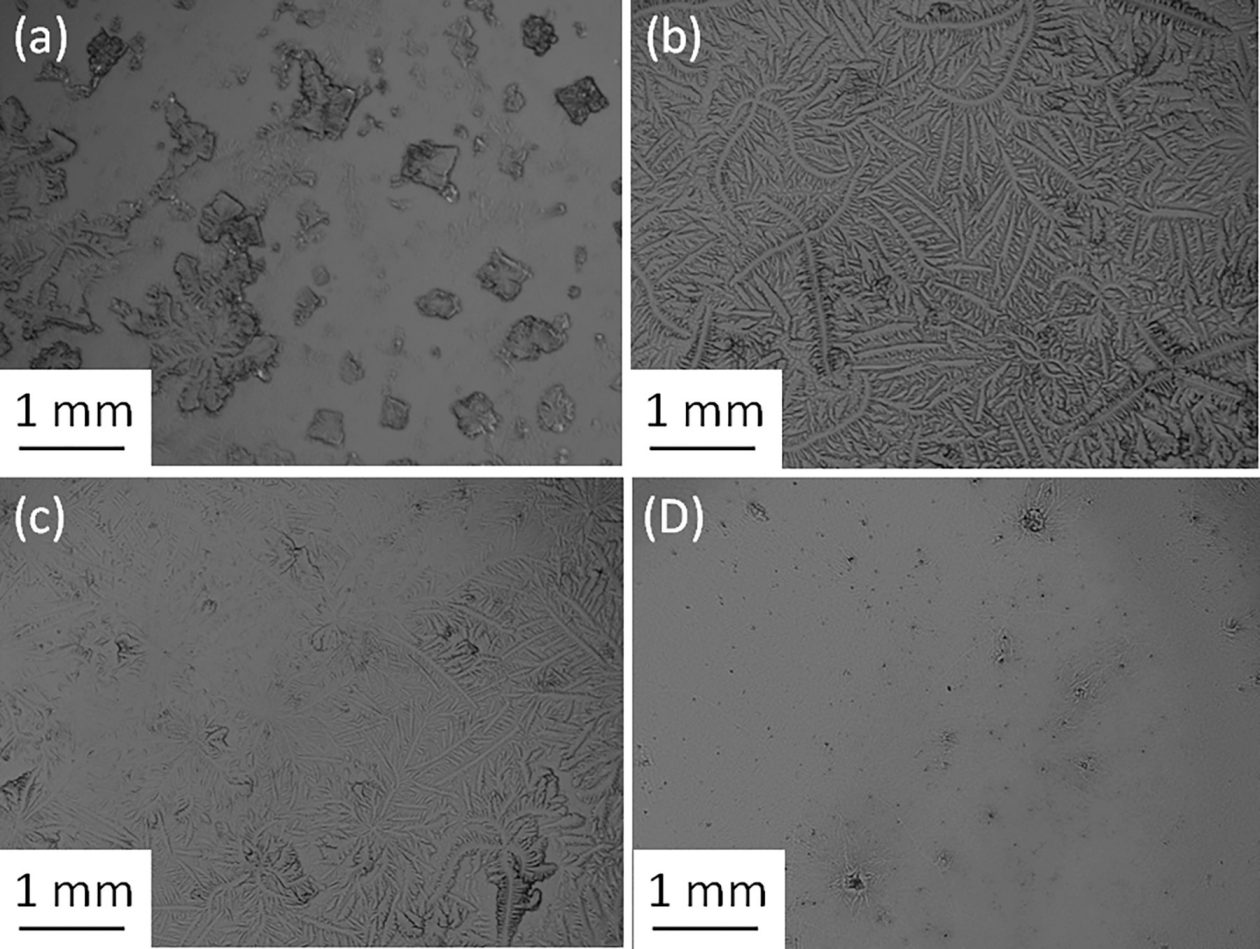
(a) Pure tears from subject 9, (b) pure tears + NaCl (20 mg, 1:3), (c) pure tears + KCl (20 mg, 1:5), and (d) pure tears + KCl (30 mg, 1:5).

**Fig 2:**
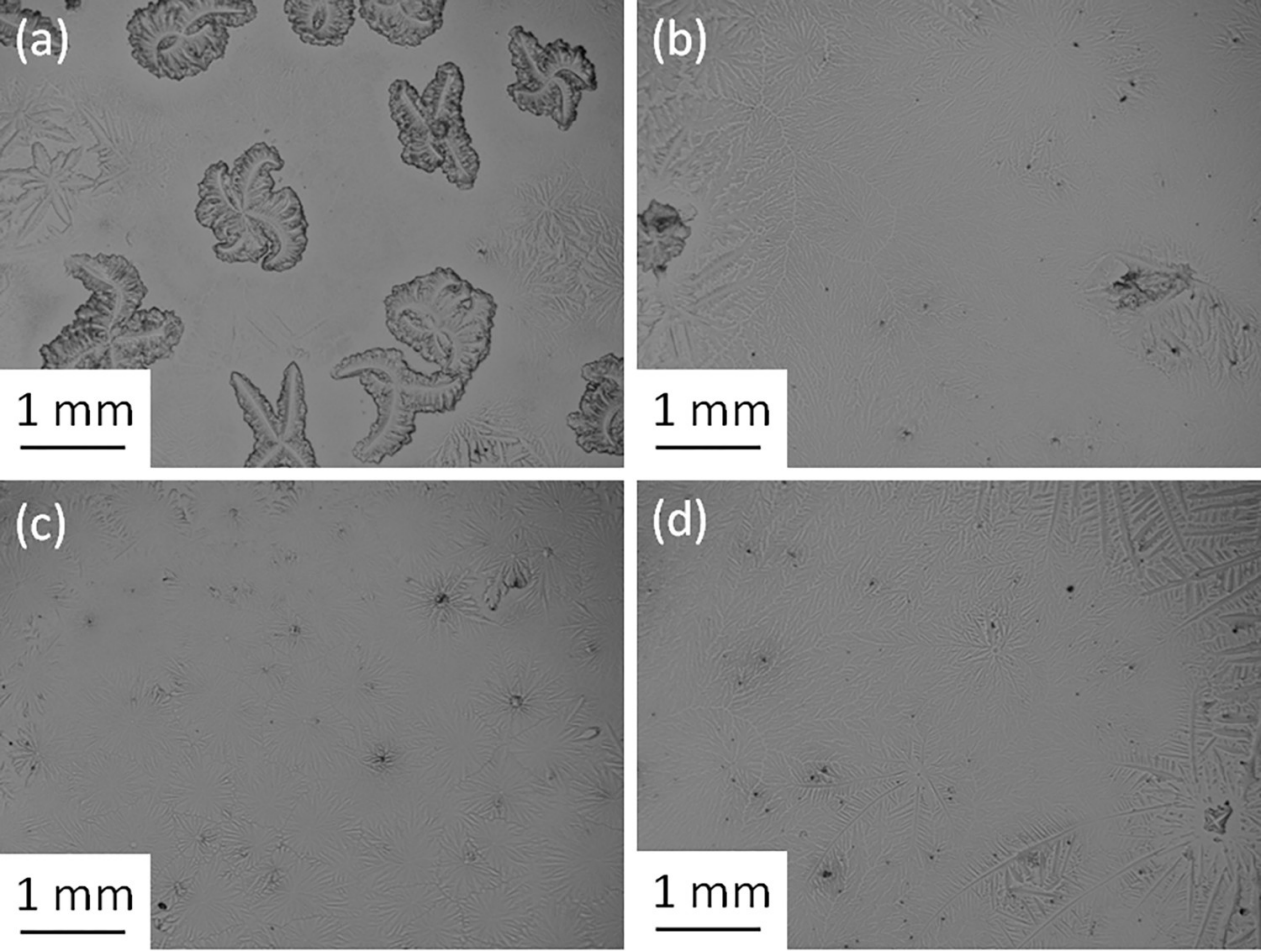
(a) Pure tears from subject 26, (b) pure tears + KCl (10 mg, 1:5), (c) pure tears + NaCl (20 mg, 1:3), and pure tears + KCl (30 mg, 1:5).

**Fig 3:**
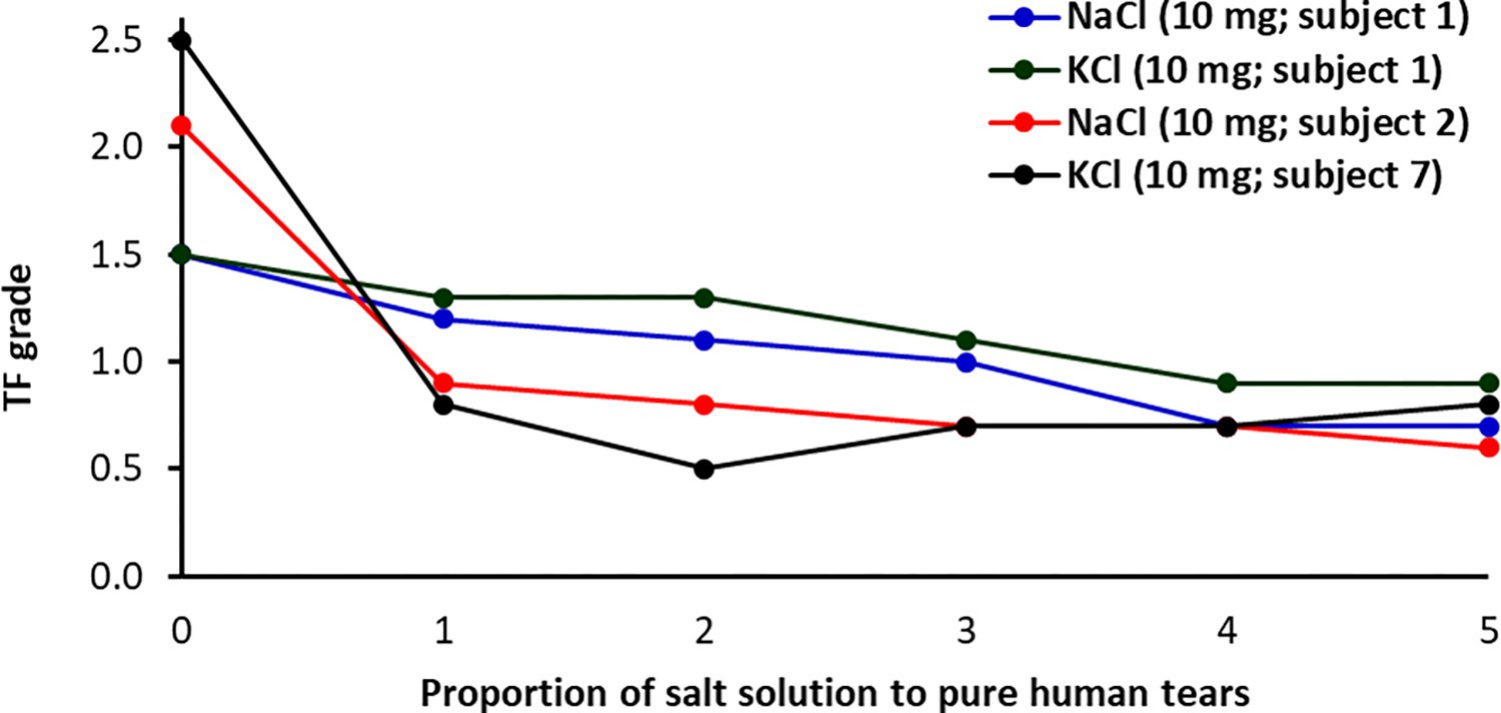
Effect of volume of NaCl and KCl solutions on the TF grade of tears collected from subjects 1, 2, and 7.

The TF patterns of pure tears collected from healthy (*N* = 13) and dry eye participants (*N* = 17) and those for the corresponding mixtures with electrolytes were observed and graded to 0.1 increments. Figs 1 and 2 show examples of the TF images of pure tears collected from subjects 9 and 26 and their mixtures with different proportions of electrolyte solution, respectively. The effect of the volume used from the NaCl and KCl solutions on the TF grade of tears collected from several subjects is shown in Fig 3.

It was clear that the use of different volumes of solutions containing various quantities of either NaCl or KCl leads to an improvement in the TF grades of human tears in all cases. Significant improvements (Wilcoxon test, *P* < 0.001) in the TF grades of tear samples were observed after the addition of either NaCl or KCl solution. In addition, a significant difference (Wilcoxon test, *P* = 0.016) in the TF grades was found between the addition of NaCl and KCl solutions. The most improvement in TF grades was noticeable for tears collected from subjects with dry eyes (TF ≥ 2). The TF grades of pure tears collected from dry-eye subjects ranged from 2.1 to 3.5, based on the five points grading scale, and decreased to be in the range of 0.4 to 1.6 after the addition of electrolyte solutions. For example, the TF grade of tears collected from subject 8 improved from 3.5 to 1.2 when a solution of either NaCl or KCl was added. While the TF grades of pure tears collected from normal-eye ranged from 1.2 to 1.9 and improved after the addition of electrolyte solution to be in the range of 0.4 to 1.5. For example, the TF grade of the tears collected from subject 29 improved from 1.6 to 0.5 when either NaCl or KCl was added.

## Discussion

The crystalline patterns of tears dried at normal conditions (room temperature and low humidity) are known as ferns. The shape of ferns provides important information about some biochemical processes. The formation of ferns depends on the type and concentration of electrolyte ions (e.g., Na cations and Cl anions) and large molecules (e.g., proteins) present in tears [24]. Artificial tears that contain electrolytes are used to release the symptoms of dry eye [24]. They improve the status of the ocular surface and tear film stability and overcome poor tear secretion [28].

It has been reported that the TF grades of tears collected from humans and animals and eye drops improved after the addition of electrolyte solutions [25–27]. Generally, divalent electrolytes lead to greater improvement in TF grades compared with monovalent ones [25–27]. However, no improvements were observed in the TF grades of the tears collected from sheep after the addition of solutions containing a large quantity (680 mg in 100 mL of water) of NaCl [25]. In addition, limited improvements were observed in the TF grades of sheep tears after the addition of solutions containing a large quantity of KCl (140 mg in 100 mL of water) [25]. Similar observations have been made for the TF grades of artificial tears [24] and tears collected from humans, camels, and goats [26, 27]. The current study showed a significant improvement in the TF grades of tears collected from humans after the addition of solutions containing a small quantity of NaCl and KCl solutions. A greater improvement in TF grades was observed after the addition of electrolyte solutions to the tears collected from dry-eye subjects. However, the collection of enough to perform the experiments tears is challenging and requires more than one sitting.

Clearly, the current research overcomes some of the limitations associated with the related work that was recently reported [25–27]. It seems likely that the presence of either NaCl or KCl in large quantities in solutions disrupts the normal ratio between large molecules and electrolytes. Such disturbance could lead to instability and hyperosmolarity in the tear film, therefore reducing the quality of tears and inducing symptoms of dry eye.

The mean difference of TF grades of human tears assessed in the morning, afternoon, and evening was 0.1 ± 0.4 based on the five-point grading scale [29]. In the current research, the TF grades recorded by two masked independent observers were almost similar (mean difference was less than ± 0.1), and the vibration was within the 95% limit of agreement.

The interactions of Na cations and Cl anions with macromolecules (e.g., proteins and mucins) caused the formation of ferns when tears were dried in a normal environment [30]. The ratio between monovalent (e.g., Na and K) and divalent cations (e.g., calcium and manganese) plays an important role in the formation of ferns [31]. The TF patterns of human and camel tears indicated that the concentration of K cations and Cl anions was high compared with those present in Refresh Plus eye drops [18]. K cations and Cl anions are important in maintaining the health of the tear film [18]. In addition, the balance between monovalent ions (e.g., Na, K, and Cl) has a controlling effect on fern formation as opposed to their concentration [30]. Moreover, large molecules (e.g., mucins and proteins) facilitate the formation of ferns. However, they are not part of the ferns’ structure [31, 32]. Indeed, the TF grades of artificial tears improved after the addition of large molecules such as sodium carboxymethyl cellulose [24].

It was noted that the correlation between the *in vitro* TF grades and the scores collected from other diagnostic dry-eye tests is poor since each test assesses a specific tear film parameter [33, 34]. Nevertheless, the TF test is valid and repeatable and provides a clear picture of tear quality. In recent years, the TF test has been used to evaluate the association between dry eye and several illnesses and habits [15, 17, 29, 35]. In principle, the TF test could help in the design of new artificial tears with high quality to relieve dry-eye symptoms. The repeatability of the TF gradings was determined using objective and subjective techniques. The TF grades are repeatable as the NITBUT and lipid layer thickness measurements. However, objective and subjective TF grades have no significant association with the tear film stability and comfort symptoms [14].

## Limitations of the study

The current study has some limitations. The subjects were mainly males. In addition, the number of monovalent electrolytes was limited to sodium and potassium chlorides and specific quantities of both of them were used. Therefore, future related studies should involve the use of other monovalent electrolyte solutions to investigate the effect of their addition on tears collected from both males and females in equal proportions.

## Conclusions

The TF test was used *in vitro* to assess the impact of the addition of a low concentration of sodium and potassium chloride solutions on tears collected from humans. The TF grades of human tears significantly improved after the addition of either sodium or potassium chloride solution. The mechanism for the improvement in TF grades due to the addition of electrolyte solutions must be investigated.
