# Correction: Effect of monovalent electrolyte solutions on the human tear ferning pattern

**DOI:** 10.1371/journal.pone.0317160

**Published:** 2025-01-03

**Authors:** The PLOS One Staff

There is an error in the affiliation of all authors. The correct affiliation is: Cornea Research Chair, Department of Optometry, College of Applied Medical Sciences, King Saud University, Riyadh, Saudi Arabia.

The publisher apologizes for the error.
